# Risk of fractures in patients with multiple sclerosis: record-linkage study

**DOI:** 10.1186/1471-2377-12-135

**Published:** 2012-11-05

**Authors:** Sreeram V Ramagopalan, Olena Seminog, Raphael Goldacre, Michael J Goldacre

**Affiliations:** 1 Department of Physiology, Anatomy and Genetics and Medical Research Council Functional Genomics Unit, University of Oxford, Oxford, UK; 2 Unit of Health-Care Epidemiology, Department of Public Health, University of Oxford, Oxford, UK

**Keywords:** Multiple sclerosis, Fractures, Epidemiology

## Abstract

**Background:**

Patients with multiple sclerosis (MS) have been reported to be at higher risk of fracture than other people. We sought to test this hypothesis in a large database of hospital admissions in England.

**Methods:**

We analysed a database of linked statistical records of hospital admissions and death certificates for the whole of England (1999–2010). Rate ratios for fractures were determined, comparing fracture rates in a cohort of all people in England admitted with MS and rates in a comparison cohort.

**Results:**

Significantly elevated risk for all fractures was found in patients with MS (rate ratio (RR) = 1.99, 95% confidence interval (CI) = 1.93-2.05)). Risks were particularly high for femoral fractures (femoral neck fracture RR = 2.79 (2.65-2.93); femoral shaft fracture RR 6.69 (6.12-7.29)), and fractures of the tibia or ankle RR = 2.81 (2.66-2.96).

**Conclusions:**

Patients with MS have an increased risk of fractures. Caregivers should aim to optimize bone health in MS patients.

## Background

Multiple sclerosis (MS) is a common, complex, immune-mediated disorder of the central nervous system (CNS) characterised by myelin loss, varying degrees of axonal pathology and progressive neurological dysfunction [1].

Patients with MS may be at risk of fracture due to low bone mineral density [2] and an elevated risk of falling [3]. Osteoporosis occurs more frequently among patients with MS, possibly as a result of immobility, imbalance, progressive disability or other undetermined yet factors [4]. An increased risk of falling may arise from imbalance or disability [3].

Previous studies have investigated the risk of fracture in patients with MS [5]. The Danish MS register found a 40% increased risk [6] and a study from the United Kingdom (UK) using the General Practice Research Database (GPRD) observed a 20% elevated risk for all fractures [7]. To investigate the association further, we undertook a record linkage study to determine the risk of fracture in individuals with MS using an English national linked Hospital Episode Statistics (HES) dataset.

## Methods

### Population and data

We used the complete dataset of English national Hospital Episode Statistics (HES) (1999–2010) covering the population of England of about 50 million people. Death data derive from death certificates. The dataset used in this study, in which successive records for each individual were linked together, was constructed by the Oxford record linkage group. The data used are not publicly available. The dataset of Hospital Episode Statistics was provided by the NHS Health and Social Care Information Centre; the mortality data were provided by the Office for National Statistics; and approval to analyse the datasets used in this study was given by the Central and South Bristol Research Ethics Committee (REC number 04/Q2006/176).

A cohort of people with a hospital admission or record of day case care for MS was constructed for those with a diagnosis of MS, as a reason for hospital care, by identifying the first admission, or episode of day case care, for the condition in a National Health Service hospital during the study period of 1999 to 2010. The International Classification of Disease (ICD) code used for MS was G35. The ICD codes used for fracture were S020, S029, S120-S129, S220-S229, S320-S329, S420-S429, S520-S529, S620-S628, S720-S729, S820-S829, S920-S929, T020-T029, T080-T089, T100 and T120 in the tenth revision; and equivalent codes for the ninth. A reference cohort was constructed by identifying the first admission for each individual with various other, mainly minor medical and surgical conditions (see Table, footnote), as used by us in other studies of associations between diseases [8,9]. In its design, the standard epidemiological practice was followed, when hospital controls are used, of using a diverse range of conditions, rather than relying on a narrow range (in case the latter are themselves atypical in their risk of subsequent disease). People were excluded from the MS or reference cohort if they had an admission for fracture either before or at the same time as the admission for MS or the reference condition.

We then searched the linked database for any subsequent NHS hospital care for, or death from, fracture in these cohorts. We considered that rates of fracture in the reference cohort would approximate those in the general population of England while allowing for migration in and out of it (data on migration of individuals were not available).

### Statistical methods

We calculated rates of fracture based on person-years. For each fracture site separately, we took “date of entry” into each cohort as the date of first admission for MS, or reference condition, and “date of exit” as the date of first record of each type of fracture, death, or the end of data collection (31st December 2010), whichever was the earliest. We first calculated rates for each fracture, stratified and then standardised by age (in five-year age groups), sex, calendar year of first recorded admission, region of residence, and quintile of patients’ Index of Deprivation score (as a measure of socio-economic status). We used the indirect method of standardisation, using the combined MS and reference cohorts as the standard population. The stratum-specific rates in the combined MS and reference cohorts were then applied to the number of people in each stratum in the MS cohort, separately, and then to those in the reference cohort, to give an observed (O) and expected (E) number of people with each fracture in each of the two cohorts. The ratio of the standardised rate of occurrence of fracture in the MS cohort was calculated relative to that in the reference cohort using the formula (O^MS^/E^MS^)/(O^ref^/E^ref^). The confidence interval for the rate ratio and χ^2^ statistics for its significance were calculated as described elsewhere [10]. The analyses were run using a suite of programs developed ‘in house’ using SAS 9 software (SAS Institute, Cary, NC, USA). We have given exact p values so that readers can judge what level of statistical significance each finding has. A full Bonferroni correction for testing multiple comparisons across eleven fracture sites would take as significant an exact p value of 0.004 (i.e. p = 0.05/11).

## Results

Table 1 shows the number of people in the study who were admitted to hospital with MS, in each age group, and it shows the percentage in each group who were female. It also shows the matching ratio, expressed as the number of controls in each age group per individual with MS in the group. All eligible controls were used within each age group to give ‘expected’ numbers of each fracture within each age group with which the observed numbers within the age group were compared (given the use of age-standardisation, nothing is gained by discarding controls to try to give equal matching ratios in each age group). Numbers of cases of MS in people under 35 years of age were very small (though numbers of controls were not); accordingly, the calculations of the observed numbers and expected numbers of each fracture within these age strata were also very small and added little to the overall rate ratio. The matching ratios within age strata showed a minimum of 36 controls per person with MS, i.e. ample numbers of controls.

**Table 1:** Number of people admitted to hospital for multiple sclerosis by age group, percentage female, and the matching ratio which gives the number of people in the reference cohort† in each age group per person with multiple sclerosis in the same age group‡

**Age group (years)**	**Number of people**	**Female (%)**	**Matching ratio**
<15	82	56.1	15615
15-24	1942	69.5	511
25-34	9003	71.0	139
35-44	17524	70.4	69
45-54	22398	70.1	36
55-64	18957	66.2	41
65-74	11296	67.1	63
75+	6671	71.7	123
Total	87873	69.1	89

† Conditions used in the reference cohort, with Office of Population, Censuses and Surveys (OPCS) code edition 4 for operations and ICD-10 code for diagnosis: appendectomy (OPCS4 H01-H03); bunion (M20.1); cataract (H25); deflected nasal septum and nasal polyp (J33, J34.2); dilation and curettage (OPCS Q10-Q11); haemorrhoids (I84); impacted tooth and other disorders of teeth (K00-K03); in-growing toenail and other diseases of nail (L60); inguinal hernia (K40); internal derangement of knee (M23); otitis externa and otitis media (H60-H67); sebaceous cyst (L72.1); squint (H49-H51); tonsillectomy and adenoidectomy (OPCS4 E20, F34, F36); upper respiratory tract infections (J00-J06); varicose veins (I83); vasectomy, female sterilisation and other contraceptive management (Z30).

‡ For example, in the age group 15–24, there are 992362 control people (511 controls for each of the 1942 people with MS in that age group). In the age group 45–54 there are 806328 controls (36 controls for each of the 22398 people with MS in that age group).

The rate ratios for fractures in England are shown in Table 2. There was an elevated risk for all fractures in MS patients compared with controls (rate ratio (RR) = 1.99, 95% confidence interval (CI) = 1.93 and 2.05). Analysis of site of fracture showed that there were elevated risks for all sites except forearm (RR = 0.91, 0.82-1.00) and wrist/hand (RR = 0.83, 0.71-0.98). Risks were particularly high for fractured neck of femur (RR = 2.79, 2.65-2.93), other femoral fracture (RR = 6.69, 6.12-7.29), and fractures of tibia or ankle (RR = 2.81, 2.66-2.96).

**Table 2:** Rate ratios and 95% confidence intervals (CIs) for fractures in people with multiple sclerosis compared with the reference cohort

				
**Fracture (ICD code**†**)**	**Observed**	**Expected**	**Rate Ratio (95% confidence interval)**	**Exact P value**
All fractures‡	4414	2238.3	1.99 (1.93-2.05)	<0.001*
Ribs (S22.2-S22.4 )	161	130	1.24 (1.06-1.45)	0.007
Clavicle (S42.0)	83	52.6	1.59 (1.26-1.97)	<0.001*
Humerus (S42.2-S42.4, S42.7)	415	204.2	2.05 (1.86-2.26)	<0.001*
Forearm (S52)	448	493.5	0.91 (0.82-1.00)	0.042
Wrist/Hand (S62)	157	188.1	0.83 (0.71-0.98)	0.025
Pelvis/Lumbar spine (S32.0-S32.8)	293	187.7	1.57 (1.39-1.76)	<0.001*
Tibia/Ankle (S82)	1393	506.1	2.81 (2.66-2.96)	<0.001*
Foot (S92)	194	95.5	2.05 (1.77-2.37)	<0.001*
Femur - neck of (S72.0-S72.2)	1579	574.2	2.79 (2.65-2.93)	<0.001*
Femur - other (S72.3-S72.8)	543	85.8	6.69 (6.12-7.29)	<0.001*
Femur - unspecified (S72.9)	88	18.5	4.91 (3.92-6.08)	<0.001*
***Fractures up to 1 year after first MS admission***	**Observed**	**Expected**	**Rate Ratio (95% confidence interval)**	**Exact P value**
All fractures‡	717	373.4	1.94 (1.80-2.08)	<0.001*
Ribs (S22.2-S22.4)	22	20.2	1.09 (0.68-1.65)	0.779
Clavicle (S42.0)	11	7.7	1.43 (0.71-2.57)	0.319
Humerus (S42.2-S42.4, S42.7)	76	28.3	2.72 (2.14-3.41)	<0.001*
Forearm (S52)	73	69.9	1.05 (0.82-1.32)	0.752
Wrist/Hand (S62)	22	28.2	0.78 (0.49-1.18)	0.278
Pelvis/Lumbar spine (S32.0-S32.8)	51	26.5	1.94 (1.44-2.56)	<0.001*
Tibia/Ankle (S82)	200	80.5	2.53 (2.19-2.91)	<0.001*
Foot (S92)	17	15.5	1.10 (0.64-1.77)	0.793
Femur - neck of (S72.0-S72.2)	293	88.2	3.39 (3.01-3.81)	<0.001*
Femur - other (S72.3-S72.8)	58	12.3	4.88 (3.68-6.35)	<0.001*
Femur - unspecified (S72.9)	18	2.7	6.91 (4.04-11.09)	<0.001*
***Fractures excluding those occurring in the first year after first MS admission***	**Observed**	**Expected**	**Rate Ratio (95% confidence interval)**	**Exact P value**
All fractures‡	3697	1864.9	2.00 (1.93-2.07)	<0.001*
Ribs (S22.2-S22.4)	139	109.7	1.27 (1.07-1.50)	0.006
Clavicle (S42.0)	72	44.8	1.61 (1.26-2.04)	<0.001*
Humerus (S42.2-S42.4, S42.7)	339	175.8	1.95 (1.74-2.17)	<0.001*
Forearm (S52)	375	423.6	0.88 (0.80-0.98)	0.019
Wrist/Hand (S62)	135	159.9	0.84 (0.71-1.00)	0.053
Pelvis/Lumbar spine (S32.0-S32.8)	242	161.1	1.51 (1.32-1.71)	<0.001*
Tibia/Ankle (S82)	1193	425.6	2.86 (2.70-3.03)	<0.001*
Foot (S92)	177	80.1	2.24 (1.92-2.60)	<0.001*
Femur - neck of (S72.0-S72.2)	1286	486	2.68 (2.54-2.84)	<0.001*
Femur - other (S72.3-S72.8)	485	73.5	7.00 (6.37-7.67)	<0.001*
Femur - unspecified (S72.9)	70	15.8	4.57 (3.55-5.81)	<0.001*

* Significant P value after full Bonferroni correction = <0.004 (most of the high rate ratios, but none of the low rate ratios, are significant on this criterion).

† Only ICD-10 codes are shown; equivalent ICD-9 codes were also used in the analysis.

‡ ICD-10 codes: S02, S12, S22, S32, S42, S52, S62, S72, S82, S92, T08, T10, T12.

Considering fractures that occurred up to a year after the first recorded admission for MS, which may reflect fractures resulting from an MS relapse or treatment, there was a significantly increased risk for all fractures combined (RR = 1.94, 1.80-2.08). However, when investigating specific fractures, elevated risks were present only for fractures of the femur, hip, humerus and tibia/ankle. There were reduced risks for fractures of the ribs, clavicle and forearm.

Considering fractures that occurred at least a year after admission for MS, there was a significantly increased risk for all fractures combined (RR = 2.00, 95% CI = 1.93-2.07). When investigating specific fractures, elevated risks were seen for all types except forearm and wrist/hand fractures.

## Discussion

Previous studies have highlighted an increased risk of fractures in patients with MS [6,7].

These previous findings, combined with our own, suggest that there is a general association between MS and the risk of fractures. There are various potential explanations for this. It has been shown that MS patients are at an increased risk of osteoporosis. This may be due to their immobility, the use of glucocorticoids or low vitamin D levels [4]. We, like others, observed substantially elevated risks for fractures of the femur, hip and tibia/ankle, which are typically related to immobility [6]. For example, our rate ratio for fractured neck of femur was similar to that in the Danish MS cohort (2.8 and 3.2, respectively), as was our rate for other femoral fracture (English and Danish figures were both 6.7). The increased risk for all fractures is present both before and after the first year following MS admission making glucocorticoid use unlikely to be the major cause of the elevated risk. Studies consistently show that bone mineral density (BMD) at the femoral neck decreases with increasing MS-related disability [11]. However, lower than expected BMD has recently been reported in individuals newly diagnosed with MS or clinically isolated syndromes as compared to controls [12] questioning a predominant role for disability. Our finding of an increased risk of fracture within the first year of admission and also years afterwards may support this but we do not have data on disability at admission to appropriately address this.

A key strength of the dataset is its size and the fact that each individual fracture site could be studied. The risk of fractures was studied all within a single population, using the same methodology, which means that direct comparisons of risk across fracture sites can be made. The dataset has limitations. The data is based on prevalent cases of MS – the first recorded hospital admission or episode of day case care for each person with MS – rather than being a cohort with follow-up from the date of first diagnosis. The dataset is limited to people who were admitted to hospital, or who received day case specialist care. We lack treatment and disability data for MS; these can themselves influence the risk of fracture. Thus the generalisability of our findings to the entire MS patient population is unclear. There is very limited information on potential confounding factors such as detailed socioeconomic characteristics and ethnicity. The effect of making multiple comparisons across different fracture sites also needs to be considered, but findings where the significance level is <0.001 or less are unlikely to be attributable to chance alone.

## Conclusion

In summary, we found a significantly elevated risk for fractures in patients with MS. Many patients with MS do not take supplemental calcium or vitamin D. Although any beneficial effect of doing so would need formally to be tested, this might be a potential area of improvement in care to optimize bone health in patients with MS [13].

## Competing interests

The authors declare that they have no competing interests.

## Authors’ contributions

MJG is the guarantor of and designed the study. SVR and MJG conceived the study. OS and RG led the analysis. SVR and MJG contributed to the analysis and interpretation of the data. SVR wrote the first draft and all contributed to subsequent drafts and the final paper. All authors read and approved the final manuscript.

## Pre-publication history

The pre-publication history for this paper can be accessed here:

http://www.biomedcentral.com/1471-2377/12/135/prepub
